# The Palliative Radiotherapy and Inflammation Study (PRAIS) ***-*** protocol for a longitudinal observational multicenter study on patients with cancer induced bone pain

**DOI:** 10.1186/s12904-018-0362-9

**Published:** 2018-09-28

**Authors:** Ragnhild Habberstad, Trude Camilla Salvesen Frøseth, Nina Aass, Tatiana Abramova, Theo Baas, Siri Tessem Mørkeset, Augusto Caraceni, Barry Laird, Jason W Boland, Romina Rossi, Elena Garcia-Alonso, Hanne Stensheim, Jon Håvard Loge, Marianne Jensen Hjermstad, Ellen Bjerkeset, Asta Bye, Jo-Åsmund Lund, Tora Skeidsvoll Solheim, Ola Magne Vagnildhaug, Cinzia Brunelli, Jan Kristian Damås, Tom Eirik Mollnes, Stein Kaasa, Pål Klepstad

**Affiliations:** 1 European Palliative Care Research Centre (PRC), Department of Clinical and Molecular Medicine, Faculty of Medicine and Health Sciences, NTNU, Norwegian University of Science and Technology and St. Olavs hospital, Trondheim University Hospital, Trondheim, Norway; 2 Cancer Clinic, St. Olavs hospital, Trondheim University Hospital, Trondheim, Norway; 3 European Palliative Care Research Centre (PRC), Department of Oncology, Oslo University Hospital, and Institute of Clinical Medicine, University of Oslo, Oslo, Norway; 4 University of Oslo and Department of Oncology, Oslo University Hospital and University of Oslo, Oslo, Norway; 5 Department Oncology, Ålesund Hospital, Møre and Romsdal Hospital Trust, Ålesund, Norway; 6 Palliative Care, Pain Therapy and Rehabilitation Unit, Fondazione IRCCS Istituto Nazionale dei Tumori, Milan, Italy; 7 Edinburgh Cancer Research Centre, University of Edinburgh, Edinburgh, UK; 8 Wolfson Palliative Care Research Centre, Hull York Medical School, University of Hull, Hull, UK; 9 Palliative Care Unit, Istituto Scientifico Romagnolo per lo Studio e la Cura dei Tumori (IRST) IRCCS, Meldola, Italy; 10 Radiation Oncology Department Arnau de Vilanova University Hospital, IRB, Lleida, Spain; 11 Cancer Registry of Norway, Institute of Populationbased Cancer Research, Oslo, Norway; 12 Centre of Molecular Inflammation Research, Department of Cancer Research and Molecular Medicine, Norwegian University of Science and Technology, Trondheim, Norway; 13 Department of Infectious Diseases, St. Olav’s Hospital, Trondheim, Norway; 14 KG Jebsen Inflammation Research Center, Department of Immunology, Oslo University Hospital, Oslo, Norway; 15 Research Laboratory, Nordland Hospital, Bodø, Norway; 16 KG Jebsen Thrombosis Research and Expertise Center, Faculty of Health Sciences, University of Tromsø, Tromsø, Norway; 17 Department of Circulation and Medical Imaging, Norwegian University of Science and Technology (NTNU), Trondheim, Norway; 18 Department of Anesthesiology and Intensive Care Medicine, St Olavs Hospital, Trondheim University Hospital, Trondheim, Norway

**Keywords:** Cancer, Radiation therapy, Palliative, Bone metastases, Pain, Depression, Cachexia, Inflammation

## Abstract

**Background:**

Radiation therapy (RT) results in pain relief for about 6 of 10 patients with cancer induced bone pain (CIBP) caused by bone metastases. The high number of non-responders, the long median time from RT to pain response and the risk of adverse effects, makes it important to determine predictors of treatment response. Clinical features such as cancer type, performance status and pain intensity, and biomarkers for osteoclast activity are proposed as predictors of response to RT. However, results are inconsistent and there is a need for better predictors of RT response. A similar argument can be stated for the development of cachexia; there are currently no predictors that can identify patients who will develop cachexia later in the cancer disease trajectory. Experimental and preclinical studies show that pain, depression and cachexia are related to inflammation. However, it is not known if inflammatory biomarkers can predict CIBP, depression or development of cachexia.

**Methods:**

This multicenter, multinational longitudinal observational study will include 600 adult patients receiving RT for CIBP. Demographic data, clinical variables, osteoclast and inflammatory biomarkers will be assessed before start of RT, and 3, 8, 16, 24 and 52 weeks after last course of RT. The primary aim of the study is to identify potential predictors for pain relief from RT. Secondary aims are to explore potential predictors for development of cachexia, the longitudinal relationship between pain intensity and depression, and if inflammatory biomarkers are associated with changes in pain intensity, cachexia and depression during one-year follow up.

**Discussion:**

The immediate clinical implication of the PRAIS study is to identify potential predictive factors for a RT response on CIBP, and thereby reduce non-efficacious RT. Patient benefits are fewer hospital visits, reduced risk of adverse effects and more individualized pain treatment. The long-term clinical implication of the PRAIS study is to improve the knowledge about inflammation in relation to CIBP, cachexia and depression and potentially identify associations and mechanisms that can be targeted for treatment.

**Trial registration:**

ClinicalTrials.gov NCT02107664, date of registration April 8, 2014 (retrospectively registered).

**Trial sponsor:**

The European Palliative Care Research Centre (PRC), Department of Clinical and Molecular Medicine, NTNU, Faculty of medicine and Health Sciences, Trondheim, N-7491, Norway.

## Background

### Cancer induced bone pain (CIBP)

Pain is one of the most frequent symptoms among cancer patients [1]. About half of cancer patients with pain are not adequately treated [2, 3]. Pain and other symptoms represent a heavy burden on the patients, their families and also on the health care system. Bone metastases account for up to 70% of cancer related pain [4, 5].

Cancer induced bone pain (CIBP) often present as a combination of somatic and neuropathic pain [6]. When tumor masses expand inside bone it can cause pain by stretching of the periosteum, fractures or invasion of sensory nerves [7]. Tumor cells also release pro-inflammatory cytokines, nerve growth factors and increase oxidative stress in the bone microenvironment. The alternation of biological substances in the bone microenvironment influence pain perception by activation of glia cells, sensitization of somatic nerve endings and disruption of the normal bone homeostasis with a balanced osteoclast and osteoblast activity [8]. Secretion of biological substances from tumor cells may also influence the central nervous system and trough immune modulation change perception of pain and the efficacy of opioids [9].

### Prediction of RT response in patients with CIBP

Palliative radiation therapy (RT) is a major treatment modality for cancer induced bone pain (CIBP), often together with opioids and co-analgesics [10]. Unfortunately, not all patients experience pain relief after RT. Complete pain response is reported in about one quarter of the patients, and partial pain response in 40–60% [11, 12]. Median time to pain response is 1 to 4 weeks [13]. RT are in most cases not associated with severe adverse effects, but nausea and vomiting is reported in up to 77% of patients [14], and pain-flares during and after RT is reported by up to one third of the patients [15, 16]. Diarrhoea, skin reactions, lethargy and tiredness are also potential adverse effects after RT [11].

Based on a relative high number of patients not responding to RT, time to treatment effect and the risk of potential side effects it is important to identify potential predictors for RT response. Former RT trials have used different pain assessments and different definitions for RT response. An international consensus on endpoints in RT trials were first published by Chow et al. in 2002 and updated in 2012 and have resulted in more comparable RT trials [17, 18]. Two recent papers on rather large patient materials found that patients with breast or prostate cancer and higher performance status were more likely to respond to RT. High baseline pain scores, the use of morphine, absence of visceral metastases and younger age were also associated with RT response in one out of the two papers [19, 20].

Imaging techniques may also be helpful to predict RT response. Two studies have demonstrated that low uptake on FDG PET-CT before RT predicted better RT response [21, 22]. MRI have also been proposed to predict RT response, but the published results are so far inconsistent [23, 24]. In addition both PET and MRI is time and economically difficult to establish as routine care. Biomarkers such as urinary osteoclast markers have been proposed to predict RT response in patients with bone metastases [25, 26], but this association is not present in all trials [27]. Elevated C-reactive protein (CRP) levels are related to more advanced disease in cancer patients that undergo palliative RT, but as far as we know no studies demonstrate a relationship between the level of CRP or other inflammatory markers and RT response in patients with CIBP [28]. Previous published literature on clinical predictors for response to RT has also no information about radiographic appearance of metastases like sclerotic/osteolytic metastases, soft tissue expansion outside bone, other cancer related symptoms or biomarkers as potential predictors.

Thus, there is still not enough information to predict which patients who are likely to respond poorly, and consequently should not receive RT. To identify patients who have increased likelihood of successful analgesia from palliative RT would improve cancer pain management.

### Cancer related symptoms and the role of inflammation

In recent years there has been an increased interest among researchers to understand the correlation between the immune system and development of cancer and cancer-related symptoms. Inflammation is present in the majority of patients with advanced cancer [29], and it is therefore difficult in cross sectional studies to establish if inflammation is simply associated with severity of the cancer disease, or whether inflammation itself elicits symptoms and can be a target to reduce symptoms like pain, depression or cachexia [30].

#### Cancer induced bone pain and the role of inflammation

Based on results from pre-clinical studies several inflammatory mediators are proposed to play an important role in CIBP. The pro-inflammatory interleukin (IL)-1β, IL-6 and tumor necrosis factor (TNF)-α are associated with nociception in animal models of bone cancer [31, 32]. Other inflammatory mediators, including chemokines such monocyte chemoattractant protein (MCP)-1, macrophage inflammatory protein (MIP-1)α, and the anti-inflammatory cytokine transforming growth factor (TGF)β, are upregulated in animal models of bone metastases, and receptors for cytokines are also present on the osteoclast surface. The release of inflammatory cytokines from tumor cells may therefore contribute to osteoclast activation with CIBP as a consequense [8, 33]. Some studies have demonstrated a potential role of inflammation (CRP, IL-6) for general cancer pain [34–36]. but as far as we know there are no published clinical trials on inflammation in relation to CIBP.

#### Development of cachexia and the role of inflammation

Up to 80% of patients with advanced metastatic cancer develop cachexia, and approximately 20 % of cancer deaths are attributed to cancer cachexia [37]. Cancer cachexia has a significant impact on patient morbidity as these patients often have reduced physical function, increased fatigue and psychosocial distress. According to a recent international consensus cachexia has been defined as a multifactorial syndrome characterized by an ongoing loss of skeletal muscle mass (with or without loss of fat mass) that cannot be fully reversed by conventional nutritional support and leads to progressive functional impairment [38]. In the consensus document, cachexia is described as a trajectory from early cachexia to late cachexia, and treatment should be assigned to patients according to the different stages. A longitudinal observational study is required to establish which factors predict both the cachexia syndrome and late cachexia.

The complete pathophysiology behind cachexia is still unclear. Most studies on molecular mechanisms have been performed on different animal models; studies in humans are scarce. Anemia, low serum albumin and increased inflammatory biomarkers such as CRP, TNF-α, soluble TNF-receptor 1 (sTNF-R1), IL-1, IL-6 and interferon (IFN)-gamma are observed in patients with cachexia [39]. Inflammatory markers such as TGFβ involved in development of bone metastases is probably also important in development of cachexia [40]. However, systemic inflammation is seen in a majority of patients with cancer, and not all of them develop cachexia therefore as for pain longitudinal studies are needed.

#### Depression in patients with advanced cancer and the role of inflammation

Depression disorder contribute to reduced quality of life in patients with advanced cancer [41]. Depression disorder is common among cancer patients with a prevalence that varies from 5 to 30% between studies [42]. A particular challenge for assessment of depression in patients with advanced cancer is the somatic depression symptoms (fatigue, psychomotor retardation, appetite disturbance and sleep disturbance) which constitute 4 out of 9 diagnostic criteria for depression disorder. In patients with advanced cancer the somatic depression symptoms can result from depression, the cancer, treatment effects, or a combination of these factors [43].

In a cross-sectional study that also controlled for disease load, we demonstrated that by only using the 5 psychological depression symptoms for defining depression disorder, we could circumvent the challenge of the overlap between the somatic depression symptoms and symptoms of the cancer [44] [45]. However, our study was cross-sectional thus limiting the possibility to assess causality [44]. Studying the development of depression disorder and each of the depression symptoms over time with control for disease and treatment(s) can provide evidence-based guidance on how to best assess depression in patients with advanced cancer.

Patients with advanced cancer and depression disorder have more somatic symptoms including pain compared to non-depressed patients, but no appropriately designed studies to examine a possible causal relationship have to our knowledge been published. Clinically, a common observation is that long-lasting pain is associated with increased levels of depression symptoms. Still the direction of causality is not established and might be bidirectional. The relation between pain and depression has to be studied in a longitudinal design in order to better understand how they are related.

Behavior corresponding to the depression symptoms, such as lowered mood, anhedonia, fatigue, sleep and appetite alterations, are also observed as a response to infections in mammals and then termed sickness behavior. Sickness behavior has been recognized as a symptom cluster that is associated with pro-inflammatory cytokine activation [46]. In non-cancer patients depression disorder has been linked to inflammation and oxidative stress [47]. Meta-analyses have demonstrated that IL-6, TNF, and CRP are elevated in non-cancer patients with depression disorder [48]. A limited number of studies have demonstrated increased levels of cytokines (IL-6, IL-8,TNFα) in cancer patients with depression [49, 50]. These findings need verification in studies using a validated measure of depression and controlling for disease and other factors known to affect the inflammatory response.

### The palliative radiotherapy and inflammation study (PRAIS)

As cancer treatment develops, a wider range of treatment options are available to each patient, and the need for tools to determine treatment response arises to select the right individual treatment. The primary aim of the PRAIS study is to combine clinical and biomarker predictors to predict the response to RT for patients with CIBP.

The availability and indications for new cancer therapeutics that modulates the immune system in order to delay development and even cure cancer have increased the last years, and a better understanding of the role of systemic inflammation in relation to cancer and cancer associated symptoms are of interest when the field of immunotherapy develops. To better understand complexity of systemic inflammation one need to extrapolate knowledge from pre-clinical studies to clinical studies with a robust study design. Secondary aims of the PRAIS study are, therefore, in a longitudinal study to explore the relationship between cancer related symptoms and inflammatory biomarkers during a one-year follow up.

## Methods

### Design

A multicenter, international longitudinal observational study of patients commencing palliative RT for CIBP.

### Study objectives

**Fig. 1:**
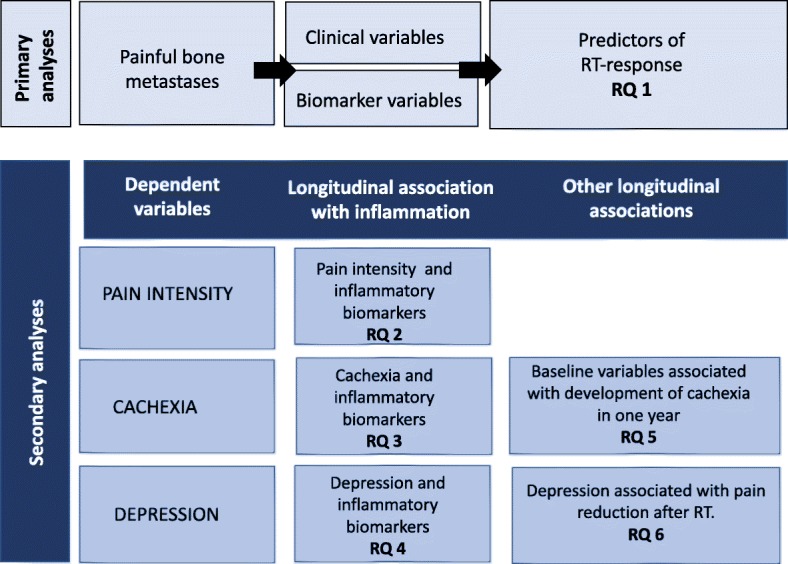
Study objectives with Research Questions (RQ)

The primary aim of the PRAIS study is to investigate clinical and biomarker predictors of pain response after palliative RT for CIBP. In the secondary analyses of this longitudinal study we will explore association between inflammation and pain intensity, cachexia, and depression in patients with CIBP that undergo palliative RT, factors associated with development of cachexia in one-year follow up and correlation between pain intensity and depression (Fig. 1).

### Study population

#### Patients to be screened for participation

All patients admitted to palliative RT for bone cancer pain is eligible for screening.

#### Inclusion criteria

- Verified cancer diagnosis, including hematological malignancies (based on radiological, histological, cytological or operative evidence)
- Bone metastases verified either by x-ray, bone scan, computer tomography (CT) or magnetic resonance imaging (MRI)
- Patients that are about to undergo RT with palliative intent for painful bone metastases
- RT to be administered within 1 week after baseline observations are obtained
- Age ≥ 18 years
- Patient able to comply with trial procedures

#### Exclusion criteria

- Pathological fracture in long bones
- Patients not consenting to participate in the study
- On-going RT or RT administered within the last 4 weeks
- Patients who are not able to follow the trial procedures
- Previous participation in this study

### Measurements

#### Timeline

The patients will be assessed at baseline and 3, 8, 16, 24 and 52 weeks after RT.

#### Clinical variables

At inclusion the following information will be collected (variables included as independent variables in the primary analyses are denoted with an asterisk): Age^*^, gender^*^, ethnicity, height, comorbidity^*^ (Charlson Comorbidity Index) [51], Karnofsky performance status^*^ [52], living situation, educational level, alcohol use, smoking status^*^, type of department (palliative care unit, surgical ward, general oncology ward, out-patient clinic, other), oncological history related to the current cancer disease (tumor diagnosis^*^, time since cancer diagnosis^*^, presence of metastases other than bone^*^, osteolytic metastasis at each planned site for RT^*^, soft tissue expansion at each planned radiation site^*^, previous and on-going anti-cancer treatment: (RT, surgery, chemotherapy, hormonal treatment, other interventions – for the primary analyses current oncological treatment other than RT (Y/N)^*^), planned RT (fractions^*^, total dose^*^ and anatomical region; narrow to radiation location in weight bearing bone (Y/N) for the primary analyses^*^, re-irradiation, previous pain treatments (duration of opioid treatment and previous unsuccessful trials with other opioids) and medications and doses for the previous 24 h (for the primary analyses we will include opioid dose in oral morphine equivalents last 24 h^*^and steroids^*^). In addition, an objective measurement of lean body mass, measured by CT scan of L3 and/or Th4 vertebrae will be performed at baseline.

At both baseline and at all follow-up visits the following self-reports will be collected: Health related quality of life (EORTC QLQ-C15 PAL) [53] (for the primary analyses trouble sleeping^*^, nausea^*^ and constipation^*^ will be included as variables), nutritional status (PG-SGA) [54], weight, overall and site specific average and worst pain last 24 h (BPI) [55] (for the primary analyses “worst pain last 24h”^*^), pain at rest and at movement from each planned irradiated site [56] (for the primary analyses we will use the variable that reflects worst pain at the irradiated site at baseline^*^) and depression^*^ (PHQ9) [57]. Specific questions related to episodic pain^*^ and self-report neuropathic symptoms and signs^*^ (LANSS) [58] will be asked at baseline and after 3 and 8 weeks.

Data on history of hospitalizations, changes in medications, new cancer treatment, treatment for hypercalcemia, new pathological fractures or spinal cord compression since last visit will be obtained at all follow-up visits (Table 1).

**Table 1:** Flow chart for registrations

	Time of assessment					
	Inclusion	3 W	8 W	16 W	24 W	1 YR
Screening for inclusion	X					
Demographics, cancer history, planned RT, previous pain therapy	X					
New cancer related incidents		X	X	X	X	X
Current use of medications	X	X	X	X	X	X
Weight	X	X	X	X	X	X
Performance status	X	X	X	X	X	X
CT scans for body composition (if available)	X	X	X	X	X	X
PG-SGA	X	X	X	X	X	X
Pain registrations	X	X	X	X	X	X
QLQ-C15 PAL	X	X	X	X	X	X
LANSS	X	X	X			
Episodic pain questions	X	X	X			
PHQ9	X	X	X	X	X	X
Blood samples for clinical chemistry	X	X	X	X	X	X
Blood samples for biomarkers	X	X	X	X	X	X
Blood samples for genetics	X					

#### Blood samples

Standard clinical chemistry will be obtained at all visits and analyzed at the local laboratory in each study center. Full blood for genetic analyses is obtained at baseline and serum for analyses on biomarkers is obtained at all visits. The serum samples will be centrifuged at room temperature at 2200 g for 10 min, frozen within 1 hour and stored at − 80 degrees Celsius until analyses. A M*ultiplex cytokine assay* (Bio-Plex Pro™ Human Cytokine Plex-27 Assay, Bio-Rad Laboratories, Hercules, CA) will be carried out to analyse inflammatory biomarkers. Cytokines involved in bone remodelling will be analysed by EIAs using matched antibodies from R&D Systems. High-sensitivity CRP will be analysed on a MODULAR platform (Roche Diagnostics, Basel, Switzerland.) For patients where collection of blood samples at follow-up is not possible (i.e. due to travelling distance from study center), the clinical data are obtained, and participation is allowed without the collection of follow-up blood samples (Table 2).

**Table 2:** Overview of blood samples obtained at all visits

Clinical chemistry	Hemoglobin, white blood cells, differential white cell count, platelets, creatinine, urea, bilirubin, potassium, sodium, chloride, total calcium, phosphate, magnesium, CRP, albumin, triglycerides, vitamin-D
Inflammatory biomarkers	High-sensitivity CRP, IL-1β, IL-1, IL-1ra, IL-2, IL-4, IL-5, IL-6, IL-7, IL-8/CXCL-8, IL-9, IL-10, IL-12 (p70), IL-13, IL-15, IL-17, basic fibroblast growth factor (bFGF), granulocyte colony-stimulating factor (G-CSF), granulocyte-macrophage colony-stimulating factor (GM-CSF), interferon-gamma (IFN-ɣ), eotaxin/CCL11, IFN-ɣ-inducible protein (IP-10)/CXCL10, MCP-1)/CCL2, MIP-1α/CCL3), MIP-1β/CCL4, regulated on activation, normal T-cell expressed and secreted (RANTES)/CCL5, TNF, platelet-derived growth factor (PDGF), and vascular endothelial growth factor (VEGF).
Biomarkers involved in bone remodelling	RANK-ligand (RANKL), Osteoprotegerin (OPG) and Notch Ligands: DLL1 and Periostin.

### Power consideration

The sample size is based upon the primary outcome; response to RT for pain [18]. The analyses plan includes up to 29 independent clinical variables (see previous sections for variables marked with an asterisk.) A full statistical estimate of sample size requires knowledge of the variance-covariance matrix, which was not available at the planning stage of this study. Therefore, the widely-used rule of thumb of 10 x number of variables was adopted. This reasoning resulted in a sample size of 290. Based upon experience, interactions will arise which increases the needed number of patients, and up to 20% of patients were expected to be lost to follow-up. To account for this, the number of needed patients was set to 600. The original protocol plan was to include a validation sample of 1/3 of patients resulting in a total number of 1000 included patients. But because of slow recruitment, we had to close the study after 600 recruited patients, and the analyses will therefore be performed without the planned validation sample.

### Trial centers

The trial centers are St. Olavs University hospital, Trondheim, Norway; Oslo University Hospital, Oslo, Norway; Ålesund Hospital, Ålesund, Norway; Fondazione IRCCS Istituto Naxionale dei Tumori, Milan, Italy; Istituto Scientifico Romagnolo per lo studio e la cura dei tumori (IRST), Meldola (FC), Italy; Hospital Universitari Arnau de Vilanova, Lleida, Spain; Castle Hill Hospital, Cottingham, United Kingdom.

### Outcome and statistical analyses

For all planned analyses baseline data will be presented with descriptive statistics; continuous variables as mean with standard deviation and categorical variables as frequency with percentages. A detailed overview of research questions and statistical analyses are described in the following section. All analyses will be performed using SPSS v 23 (IBM Corp. Armonk, NY) and STATA v 16 (Stata Corporation LP; College Station, TX, USA).

**Research question 1:** Which are the clinical predictors and biomarkers predictors of pain response to palliative RT for CIBP?

*Objective*: To obtain demographic, clinical data and biomarkers before start of RT and compare with RT response for pain in order to develop a classification system relevant for predicting RT response.

*Outcome*: Primary endpoint is response to RT defined as at pain reduction of worst pain score of two or more at the treated site on the 11-point NRS together with no increase in analgesic intake, or a reduction in opioid intake of at least 25% from baseline without an increase in worst pain score at the treated site [18]. Patients with two or more radiation locations are defined as responders if they respond in one of the included sites. Patients who die before the first assessment (3 weeks after RT), will be defined as non-responders because these patients have not benefited from RT. Patients with missing data on outcome measurements including pain intensity at the treated site or the use of analgesic medications will not be included in the analyses.

*Statistical method*: Clinical variables (see previous chapter) and CRP will be included in a multivariate logistic regression model to predict potential factors for response to RT. The model will be adjusted by study centre. Regression diagnostics will be performed for all analyses adding interactions terms if necessary. Significant variables from the multivariable model will be presented as a response-score to determine the likelihood of response to RT. Second, we will perform analyses on inflammatory biomarkers and bone biomarkers correlated with response to RT. Only inflammatory biomarkers and bone biomarkers with levels of serum concentrations above the detection threshold and which have a variability between measurements will be analysed with a multivariate logistic regression model with response to RT as the dependant variable. Finally, an integrated regression model with response to RT including both the significant clinical variables and significant biological biomarkers will be conducted if appropriate based on results from the previous analyses.

**Research question 2:** Which inflammatory biomarkers are associated with change in pain intensity in patients with CIBP?

*Objective*: To investigate the correlation between pain intensity and inflammation. Patients included in the PRAIS study all have CIBP and will receive a standardized intervention expected to give pain relief in about 60% of the patients. This study can explore if there is a longitudinal relationship between changes in pain intensity and the detected level of inflammatory biomarkers.

*Outcome*: Serum concentrations of inflammatory biomarkers as described above associated with changes in pain intensity as measured by average pain intensity and worst pain intensity last 24 h (NRS 0–11) for a maximum of 1 year follow-up.

*Statistical method*: Longitudinal data analysis with repeated measurements using a liner mixed effect model or generalized estimating equation (GEE).

**Research question 3:** Which inflammatory substances are associated with cancer cachexia?

*Objective*: To gain insight into the role of inflammation in cancer cachexia.

*Outcome*: Cachexia is defined as a) weight loss > 5% over past 6 months (in absence of simple starvation); or b) body mass index (BMI) < 20 and any degree of weight loss > 2%; or c) appendicular skeletal muscle index consistent with sarcopenia (males < 7・26 kg/m2; females < 5・45 kg/m2) and any degree of weight loss > 2% [38]. Cachexia severity is assessed as degree of weight loss and EORTC QLQ C15 PAL physical function and appetite loss.

*Statistical method*: Fist, we will perform a cross-sectional analysis in the baseline parameters using a logistic regression with cachexia as outcome, and the various inflammatory markers as explanatory variables. Second, we will investigate the association between changes in inflammatory markers over time and cachexia severity using mixed linear modelling.

**Research question 4:** Which inflammatory substances are associated with depression in cancer patients?

*Objective*: To explore the associations between serum concentrations of inflammatory substances and depression.

*Outcome*: Depression is measured by the PHQ-9 questionnaire [57]. The PHQ-9 includes 9 items identical to the diagnostic criteria (i.e. the depression symptoms) for depression disorder. For the purpose of these analyses we will use depressive disorder as defined by the PHQ-9 recommended sum score calculation, and symptom by symptom in the PHQ-9 score in associations of serum concentrations of inflammatory biomarkers during a period of 1 year form inclusion.

*Statistical method*: We will use repeated measurements and a linear mixed effect model to perform the analyses.

**Research question 5:** Which are the clinical predictors and biomarker predictors of development of cachexia in patients with metastatic cancer disease?

*Objective*: To obtain demographic and clinical data and biomarkers in a homogenous population of patients with metastatic cancer, and within a prospective, longitudinal follow-up study observe which factors predicting the development of severe cachexia during one-year follow-up.

*Outcome*: Cachexia is defined as for research question 3.

*Statistical method*: As the purpose is to evaluate development of cachexia, only patients without cachexia at baseline will be included in this analysis. A Cox regression will be used to evaluate baseline predictors of cachexia development.

**Research question 6**: What is the relationship between pain reduction and depression in patients receiving RT for CIBP?

*Objective*: RT is expected to reduce pain in a substantial number of patients. This creates a possibility to study the relationship between depression and pain longitudinally in an experimental-like design in which one variable (pain) is manipulated and the effect on another related variable (depression) is studied.

*Outcome*: Depression is measured as for Research question 4.

*Statistical method*: Longitudinal analyses with repeated measurements analyzed with a linear mixed effect model.

### Ethics

A signed consent will be obtained from all participants by an investigator at each site. The study will be carried out in accordance with ICH GCP and the World Medical Association Declaration of Helsinki (1964) and its’ revisions (Tokyo 1978, Venice 1983, Hong Kong 1989, South Africa 1996 and Edinburgh 2000). The study is approved by The Regional Committee for Medical and Health Research Ethics, REC Central Norway, and by the regulatory authorities at each trial site. If modifications to the protocol, amendments will be applied for to the regional ethics committee and after approval distributed to all local study investigators.

RT is indicated and will be given regardless of whether the patients choose to participate in the study or not. Thus, study participation means that patients consent to report symptoms and have their blood drawn. The volumes of blood samples are limited (less than 50 ml) and give no extra risk for anemia. Thus, the study includes no interventions that increase the risk for the patients. Data are handled anonymously. The database used for analyses only identify each patient by a study number. The linkage between study number and patient identity is in a document and / or memory stick stored in a safe at each study center.

### Organizational issues

A Trial Steering Group will oversee the running of the trial. Members of the trial steering group include the chief principal Investigator, the principal Investigators of each centre, the Clinical Trial coordinator, and the trial statistician. After completion of the inclusions the Trial Steering Group will consists of the chief principal investigator, the principal investigators at all sites including 100 patients or more, and the trial statistician.

The coordinating centre will administer the study. This includes development and administration of Case report forms (CRFs), monitoring of data quality and preparation of the final study report. (CRFs) will be supplied by the coordinating centre. Specific queries about data will be addressed to the clinical trial coordinator at each study centre.

The results will be published in peer-reviewed journals. Authorship is based upon the Vancouver rules. All manuscript will be prepared by the researchers. The trial steering group will make decision related to use of data for publication. The principal investigator has access to all data. Access to researchers will be decided based upon the actual need for access relevant for analyses. Access to anonymously participant-level data set will be supplied depending on the journal policies.

## Discussion

Clinical trials in cancer are mostly carried out to evaluate and compare different treatment strategies, medications and interventions. Whiles the effect of RT to treat CIBP is well documented, there is limited information about which characteristics that differs among responders versus non-responders. To achieve reliable predictors for treatment response, studies with large patient materials that obtain follow-up visits are needed. In this study, detailed clinical information, inflammatory and bone biomarkers will be collected before RT. These are all data and analyses that could be included in routine clinical practice to better select patients likely to have RT response. This can reduce the administration of non-efficacious RT. Patients will benefit from not spending time on futile treatment and not risking adverse effects related to RT as well as not delaying alternative pain treatments.

Our research group has in the last decade published several papers on cancer pain and the relationship between pain and other cancer symptoms to improve cancer pain classification [59–61]. International collaboration have also developed the Edmonton classification for cancer pain [56, 62]. The PRAIS study will include a large cancer patient population with advanced, but not terminal, cancer disease that will be used to investigate which clinical characteristics and biomarkers are associated with other cancer related symptoms. We will explore the longitudinal associations for pain, depression and cachexia with clinical characteristics, and biomarkers.

The investigation of the role of inflammation in respect to RT response for patients with CIBP and other cancer related symptoms like pain intensity, depression and cachexia is of a more explorative character. Current literature is mostly based on pre-clinical studies that needs to be investigated further in human studies. This study will include patients who all have on-going CIBP, which is a more homogenous population than usually seen in cancer pain studies. The patients will receive a standardized intervention expected to give pain relief in about 60% of the patients, and the study is therefore a unique possibility to identify which inflammatory substances that are associated with changes in cancer pain intensity. Improved knowledge about the pathophysiology of bone cancer pain and the role of inflammation can identify potential targets for therapy. The longitudinal design of the study also makes it possible to study the relationship between inflammation and symptoms of depression and development of cachexia. If biological factors are detected they could be used to identify which patients who could benefit from therapy directed towards hindering or delaying the development of severe cachexia or treatment of depression in cancer patients.

### Expected limitations

As for all studies on palliative cancer patients we expect the number of drop-outs and missing data to be relatively high. A reason for drop-out before evaluation of treatment response may be that the patients are too sick or because of high symptom burden. These patients cannot be included in the predictive analyses as they are not missing at random and may bias the results. The patient inclusion is also for practical reasons not consecutive and the sample size of 600 do not allow for a validation sample analyses. Also, in order to assess the adverse effects from RT, a day-to-day evaluation of symptoms after RT would have given important information but were not performed in order to simplify data collection for the patient population.

## Abbreviations

BPI: Brief Pain Inventory
CIBP: cancer induced bone pain
CNS: Central Nervous System
CRP: C-Reactive Protein
CT: Computer Tomography
EORTC QLQ: European Organization for Research and Treatment of Cancer Quality of Life Questionnaire
FDG- PET – CT: Fluorodeoxyglucose Positron Emission Tomography/Computed Tomography
ICH GCP: The International Council for Harmonization Guideline for Good clinical practice
IL: Interleukin
LANSS: Leeds Assessment of Neuropathic Symptoms and Signs
MCP: Monocyte Chemotactic Protein
MIP: Macrophage Inflammatory Protein
MRI: Magnetic resonance imaging
NRS: Numeric Rate Scale
PET: Positron emission tomography
PG-SGA: Patient-Generated Subjective Global Assessment
PHQ-9: The patient health questionnaire-9
RQ: Research Question
RT: Radiation therapy
sTNF-R1: Serum soluble receptor for TNF
TGF: Transforming growth factor
TNF: Tumor Necrosis Factor
